# Immune interface interference vaccines: An evolution‐informed approach to anti‐bacterial vaccine design

**DOI:** 10.1111/1751-7915.14446

**Published:** 2024-03-27

**Authors:** Nicholas J. Croucher

**Affiliations:** ^1^ MRC Centre for Global Infectious Disease Analysis, Department of Infectious Disease Epidemiology, School of Public Health Imperial College London London UK

## Abstract

Developing protein‐based vaccines against bacteria has proved much more challenging than producing similar immunisations against viruses. Currently, anti‐bacterial vaccines are designed using methods based on reverse vaccinology. These identify broadly conserved, immunogenic proteins using a combination of genomic and high‐throughput laboratory data. While this approach has successfully generated multiple rationally designed formulations that show promising immunogenicity in animal models, few have been licensed. The difficulty of inducing protective immunity in humans with such vaccines mirrors the ability of many bacteria to recolonise individuals despite recognition by natural polyvalent antibody repertoires. As bacteria express too many antigens to evade all adaptive immune responses through mutation, they must instead inhibit the efficacy of such host defences through expressing surface structures that interface with the immune system. Therefore, ‘immune interface interference’ (I3) vaccines that target these features should synergistically directly target bacteria and prevent them from inhibiting responses to other surface antigens. This approach may help us understand the efficacy of the two recently introduced immunisations against serotype B meningococci, which both target the Factor H‐binding protein (fHbp) that inhibits complement deposition on the bacterial surface. Therefore, I3 vaccine designs may help overcome the current challenges of developing protein‐based vaccines to prevent bacterial infections.

## Graphical abstract

Many bacterial pathogens express proteins and polysaccharides that inhibit host immune responses. This article proposes that immune interface interference vaccines may be an effective means of preventing bacteria from evading host defences.

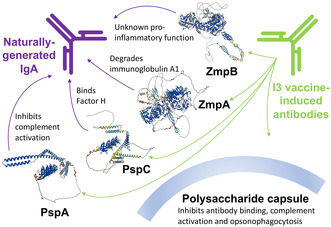

## THE CHALLENGES OF PRODUCING ANTI‐BACTERIAL VACCINES

The rapid deployment of vaccines to curtail recent outbreaks of mpox (Deputy et al., 2023), SARS‐CoV‐2 (Jackson et al., 2020) and ebola (Regules et al., 2015) highlights the speed with which new or existing vaccines can be developed, licensed and applied against many zoonotic viruses. Such pathogens exhibit limited antigenic diversity as a consequence of their recent host jump and are therefore relatively easily targeted by formulations containing one, or a few, antigens. Many such highly effective anti‐viral immunisations (Table 1) consist of comparatively simple formulations containing a single protein antigen (Jackson et al., 2020), live viruses attenuated through serial passage (Gruber, 2022), or a chemically‐inactivated preparation of virions (Braconier et al., 1999).

**TABLE 1:** A summary of the pathogens currently preventable by vaccines licensed for use in the USA (CDC, 2023).

Pathogen	Pathogen type	Vaccine type	Valency
Adenovirus	Virus	Live attenuated	Polyvalent
*Bacillus anthracis*	Bacteria	Protein (including toxin)	Monovalent
*Mycobacterium tuberculosis*	Bacteria	Live attenuated	Monovalent
Chikungunya	Virus	Live attenuated	Monovalent
*Vibrio cholerae*	Bacteria	Live attenuated	Monovalent
SARS‐CoV‐2	Virus	mRNA	Polyvalent
Dengue	Virus	Live attenuated	Polyvalent
*Corynebacterium diphtheriae*	Bacteria	Toxoid	Monovalent
*Clostridium tetani*	Bacteria	Toxoid	Monovalent
*Bordetella pertussis*	Bacteria	Protein (including toxoid)	Monovalent
Ebola	Virus	Live attenuated	Monovalent
*Haemophilus influenzae b*	Bacteria	Conjugate polysaccharide	Monovalent
Hepatitis A	Virus	Inactivated	Monovalent
Hepatitis B	Virus	Protein	Monovalent
Human papillomavirus	Virus	Protein	Polyvalent
Influenza	Virus	Inactivated or protein or live attenuated	Monovalent or polyvalent
Japanese encephalitis virus	Virus	Inactivated	Monovalent
Measles	Virus	Live attenuated	Monovalent
Mumps	Virus	Live attenuated	Monovalent
Rubella	Virus	Live attenuated	Monovalent
*Neisseria meningitidis* ACWY	Bacteria	Conjugate polysaccharide	Polyvalent
*Neisseria meningitidis* B	Bacteria	Outer membrane vesicle and/or protein	Polyvalent
*Yersinia pestis*	Bacteria	Inactivated	Monovalent
*Streptococcus pneumoniae*	Bacteria	Polysaccharide or conjugate polysaccharide	Polyvalent
Poliovirus	Virus	Inactivated	Polyvalent
Rabies	Virus	Inactivated	Monovalent
Rotavirus	Virus	Live attenuated	Monovalent or polyvalent
Respiratory syncytial virus	Virus	Protein	Monovalent or polyvalent
Mpox	Virus	Live attenuated	Monovalent
Smallpox	Virus	Live attenuated	Monovalent
Tick‐borne encephalitis	Virus	Inactivated	Monovalent
*Salmonella enterica* Typhi	Bacteria	Live attenuated or polysaccharide	Monovalent
Varicella	Virus	Live attenuated	Monovalent
Yellow fever	Virus	Live attenuated	Monovalent
Varicella zoster virus	Virus	Protein	Monovalent

*Note*: The term “live attenuated” refers to vaccines employing both replication‐competent, and non‐replicative, live components.

Multivalent vaccines have also been developed against more diverse viral pathogens (Schlingmann et al., 2018). Immunisations containing antigens enabling the targeting of serologically distinct types (serotypes) have been licensed for use against pathogens such as respiratory syncytial virus (Walsh et al., 2023) and rotavirus (Dennehy, 2008). Vaccines against the highly variable influenza virus are not only multivalent but also require frequent updates in response to surveillance data (Harding & Heaton, 2018). Nevertheless, if there is a strong match between the vaccine antigen and the viruses, these programmes are highly effective. The major viral pathogens to have eluded vaccination programmes generally represent immunologically challenging targets, such as the highly diverse respiratory viruses that cause mild infections (McLean, 2020), and the immunosuppressive, and rapidly mutating, human immunodeficiency virus (Heaton, 2020).

In contrast to the successful use of immunisations against even recently emerged viruses, we still lack effective vaccines against many endemic and common bacterial pathogens (Frost et al., 2023). Until recently, the only licensed protein‐based vaccines that have proved effective against bacterial diseases have primarily neutralised the toxins they produce (e.g. the diphtheria, tetanus and pertussis toxins) rather than mainly targeting the pathogens themselves (Osterloh, 2022). Killed, or live attenuated, whole cell vaccines have been successfully used to protect against some bacterial infections, but these have only been employed against pathogens exhibiting limited genetic diversity, such as *Mycobacterium tuberculosis*, *Salmonella enterica* Typhi and *Bacillus anthracis* (Achtman, 2008). As cholera is commonly caused by two different *Vibrio cholerae* serotypes, whole cell vaccines against this disease sometimes include two strains of this bacterium (Bi et al., 2017). Nevertheless, the efficacy of these formulations is suboptimal (Bi et al., 2017; Martinez et al., 2022), and they have not been successfully used to protect against more diverse bacterial species.

Instead, the most effective vaccines against diverse multi‐strain pathogens have been polysaccharide conjugate vaccines (PCVs), which trigger immune responses to the extracellular capsules of pathogens (Croucher et al., 2018). These have been effective at near‐eliminating disease caused by *Haemophilus influenzae* type b (Morris et al., 2008) and reducing disease caused by multiple capsule types of *Streptococcus pneumoniae* and *Neisseria meningitidis* (McIntyre et al., 2012). However, these complex formulations are expensive to manufacture and only capable of targeting a subset of the many capsule types present some species (Croucher et al., 2018). Hence, there is a continued interest in developing anti‐protein vaccines that would be universally effective against all encapsulated and unencapsulated bacteria, which could help address the growing problem of antibiotic resistance (Frost et al., 2023) while exploiting newly developed mRNA vaccine technology (Jackson et al., 2020).

## THE DEVELOPMENT AND CHALLENGES OF REVERSE VACCINOLOGY

Originally, the selection of potentially protective proteins relied on immunological assays to detect antigens that could then be identified and cloned. The advent of the genomic era made it possible to instead adopt a sequence‐driven approach. This first identified candidate proteins *in silico*, enabling them to be cloned and heterologously expressed, such that their immunogenicity could then be assayed; hence this approach was termed ‘reverse vaccinology’ (Rappuoli, 2000). This approach was soon applied to designing multivalent protein vaccines for bacterial species including *N. meningitidis* (Rappuoli, 2000), *S. pneumoniae* (Wizemann et al., 2001) and *Chlamydia pneumoniae* (Montigiani et al., 2002).

As multiple genomes became available for some bacterial species, the extensive intraspecific variability in gene content enabled the refinement of the reverse vaccinology approach to ‘pan‐genomic’ or ‘population vaccinology’ (Mora et al., 2006). This involved the identification of conserved antigens encoded by the core genome, which could provoke responses with the potential to be universally protective across all members of a species. This modified approach was soon used to identify vaccine antigens in *Streptococcus agalactiae* (Maione et al., 2005), *Streptococcus pyogenes* (Rodríguez‐Ortega et al., 2006) and *Staphylococcus aureus* (Stranger‐Jones et al., 2006), among others.

The original reverse vaccinology approach was fundamental to the successful development of the protein‐based vaccines against serotype B meningococci, first licensed in 2013 (Bexsero) and 2014 (Trumenba) (Säll et al., 2020). However, despite the hope this approach would accelerate vaccine development by at least 10 years (Rappuoli, 2000), and a large number of studies using this approach (Goodswen et al., 2023), reverse vaccinology has not thus far yielded a multiplicity of licensed vaccines. This is not a consequence of failing to identify targets that are conserved across species, immunogenic in animal models, and safe to administer to humans (Aceil & Avci, 2022; Jansen et al., 2013; Pokharel et al., 2023). Instead, multiple clinical trials have not found a substantial protective antibody response to protein‐based vaccines, as described for proposed formulations targeting *S. aureus* (Jansen et al., 2013), *S. pneumoniae* (Darrieux et al., 2015) and *S. pyogenes* (Castro & Dorfmueller, 2021).

This contrasts with PCVs, which are capable of inducing a sufficiently strong mucosal immune response to prevent colonisation by targeted capsule types, making their efficacy easier to measure (Darrieux et al., 2015). Although there is interest in using a wider variety of protein antigens as carriers for polysaccharides in PCVs (Bröker et al., 2017), the conjugation of polysaccharides to an *H. influenzae* protein in a PCV did not provide measurable protection against acute otitis media caused by unencapsulated isolates of this pathogen in a cluster‐randomised trial (Vesikari et al., 2016). Hence, while PCVs of different types and increasing valency are still being developed (Croucher et al., 2018; Frost et al., 2023), there has not been such advances in the deployment of protein‐based vaccines.

## THE NATURAL DEVELOPMENT OF ADAPTIVE IMMUNE RESPONSES TO BACTERIA

Multiple high‐throughput techniques have been developed for profiling human immune responses to pathogens. These include immunoproteomic analyses, in which human sera are used to immunoblot an electrophoretically separated bacterial proteome, followed by mass spectrometric identification of antigens (Jungblut, 2001); antigenic fingerprinting, in which peptides binding to biotinylated antibodies are selected from surface display libraries (Giefing et al., 2008); proteome arrays, in which heterologously expressed microbial proteins are printed as an array, and used to quantify antibody binding (Campo et al., 2015); and B cell repertoire sequencing, in which the genes encoding the antibodies that comprise an immune response are cloned, heterologously expressed and characterised (Babcook et al., 1996). These data have been integrated into the selection of suitably immunogenic proteins for inclusion in vaccine formulations in ‘reverse vaccinology 2.0’ (Rappuoli et al., 2016).

These methods concur that the mammalian antibody response to bacteria recognises multiple proteins (Dennehy & McClean, 2012). The use of arrays representing the panproteome of *S. pneumoniae* have demonstrated healthy American adults' responses to the bacterium target many surface antigens, including multiple proteins involved in the degradation of host structures, acquisition of nutrients, adhesion and cell wall synthesis (Croucher et al., 2017). This highly polyvalent response has also been consistently observed across an international collection of serum samples (Wilson et al., 2017). Such responses develop rapidly in infants, following only a few weeks of asymptomatic colonisation (Croucher et al., 2024). Even in neonates lacking their own endogenous responses, maternal serum and breast milk antibodies recognise a broad diversity of proteins across multiple pathogens (McGuire et al., 2021). Hence, extensive adaptive immune responses recognising many opportunistic pathogenic bacteria are ubiquitous across human populations. Therefore, the relative modest responses observed to some whole cell vaccines, even with extensive proteome arrays (Campo et al., 2018; Ndungo et al., 2018), are likely a consequence of the extensive pre‐existing antibody repertoire recognising such immunisations.

This would correspond with the limited boosting of natural antibody repertoires seen in a longitudinal study of *S. pneumoniae* colonisation and immune responses in children (Croucher et al., 2024). This demonstrated repeated re‐exposure to many proteins mainly boosted relatively weak responses in the population, but did not drive continual increases across all individuals. Hence, the level of immunoglobulin G binding to each antigen plateaued at a characteristic value that was quite consistent across the cohort. Therefore, any vaccine designed using reverse vaccinology 2.0 will likely struggle to elicit a substantial response when targeting conserved, naturally immunogenic proteins expressed by bacteria that humans routinely carry asymptomatically.

## AN EVOLUTIONARY PERSPECTIVE ON THE CHALLENGE OF ANTI‐BACTERIAL VACCINATION

Even after the development of such highly polyvalent antibody responses, individuals will frequently be recolonised by the targeted bacteria (Croucher et al., 2024). This contrasts with the protective post‐infection immune response to many viral infections (Hope & Bradley, 2021). Consequently, a virus' immunodominant antigens are often under selection to evade adaptive immunity, as exemplified by the diversification of influenza haemagglutinin and neuraminidase proteins (Treanor, 2004), or the SARS‐CoV‐2 spike protein (Plante et al., 2021). However, bacteria cannot evade the natural polyvalent immune response through changing all the recognised antigens, many of which are critical for cellular survival and replication. Correspondingly, many *S. pneumoniae* surface antigens are highly conserved (Croucher et al., 2017). Hence, common opportunistic bacterial pathogens must survive in the microbiota despite their recognition by host antibodies.

**FIGURE 1:**
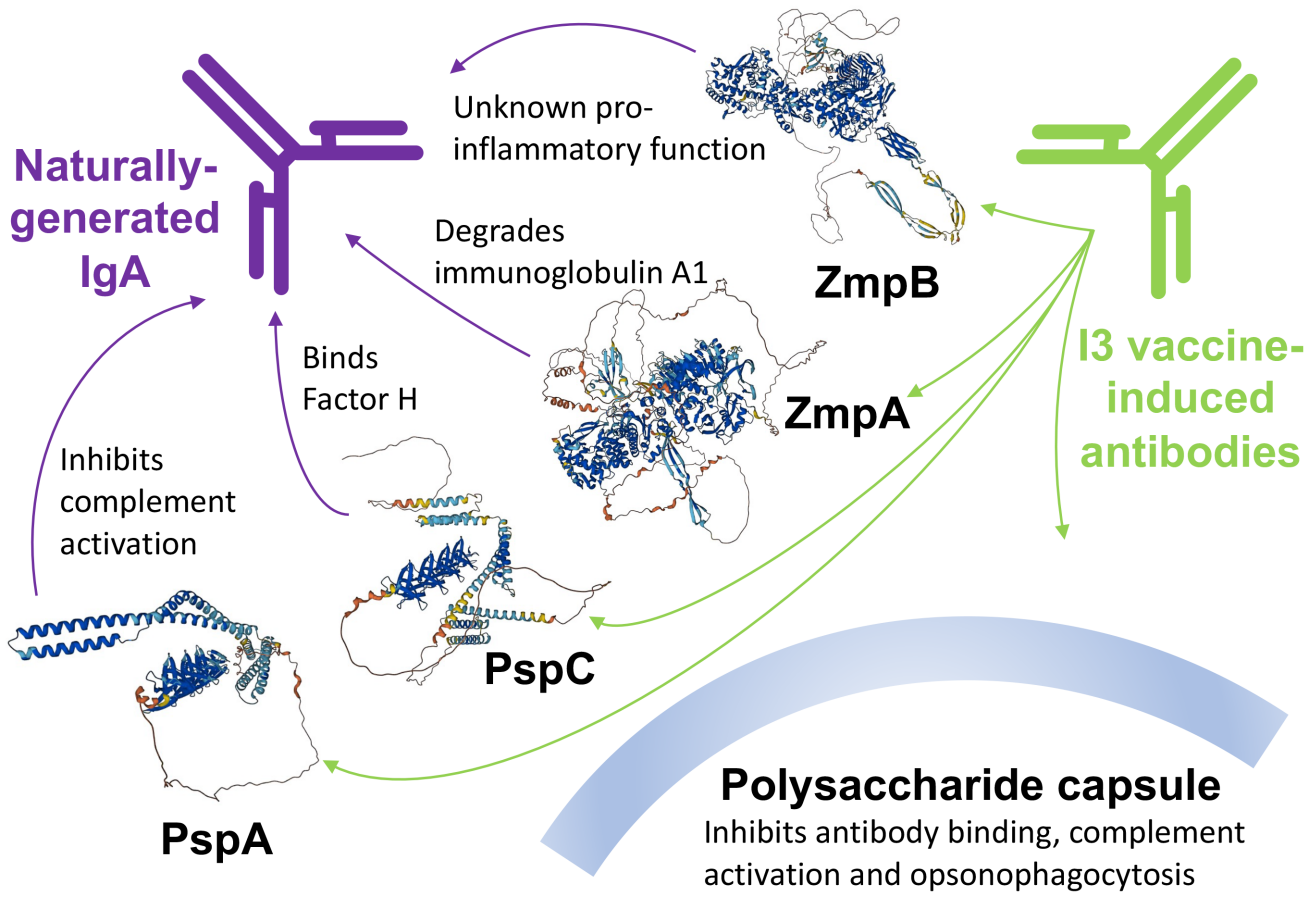
A representation of the immune interface interference (I3) vaccine design strategy applied to targeting *Streptococcus pneumoniae*. The four “diverse core loci” immune evasion proteins (PspA, PspC, ZmpA and ZmpB) are represented by their predicted structures, as accessed from the AlphaFold Protein Structure Database (Varadi et al., 2022), alongside the capsule. These bacterial factors are annotated with their immune evasion functions, as summarised in Croucher et al. (2024).

This may reflect antibody‐mediated immune responses not playing an important role in controlling bacterial growth on mucosal epithelia. Yet the evolution of these bacteria suggests otherwise. The nasopharynx is an informative example, as bacteria must remain in close association with the mucosal surfaces to survive. Common commensals of this niche have convergently evolved multiple immune evasion mechanisms that all target antibody‐mediated immune responses. Immunoglobulin A1 proteases, which degrade the most common antibody isotype in the nasopharynx, have independently evolved in *S. pneumoniae* (Bek‐Thomsen et al., 2012), *H. influenzae* (Fernaays et al., 2006) and *N. meningitidis* (Mulks & Plaut, 1978). Additionally, they each also express proteins that bind Factor H (Dave et al., 2004; Lo et al., 2009; Riesbeck, 2020), which inhibits the alternative complement cascade. *S. pneumoniae* also expresses a protein that inhibits the deposition and activation of complement on its surface (Figure 1) through an alternative mechanism (Li et al., 2007). This process is also limited by the polysaccharide capsule expressed by many isolates of all three pathogens, which additionally inhibits antibody recognition of antigens and opsonophagocytosis (Hyams et al., 2010). Notably, while the capsule is essential for causing invasive disease in all three species, the common isolation of unencapsulated isolates in carriage demonstrates it is dispensable in evading the mucosal immune system (Tsang, 2021). Hence, the activity of these immune evasion structures can both explain the ability of bacteria to infect the same host multiple times and evade the effects of any vaccine‐induced antibody responses.

Therefore, the evolutionary arms race between commensal bacteria and host immunity appears to be focussed on antibodies and the associated complement cascades. It is certainly possible that bacterial proteins could prevent other branches of the immune system, such as CD4^+^ T cells, from controlling the growth of commensal bacteria (Malley et al., 2005). However, *S. pneumoniae* comparative genomics identified just four ‘diverse core loci’ (DCL; Figure 1) that are evolving under the competing pressures of selection for the retention of their function, meaning they are ubiquitous across the species, while diversifying to avoid recognition by host adaptive immunity (Croucher et al., 2017). All four appear to function through interfering with mechanisms associated with the antibody‐mediated immune system (Croucher et al., 2024). Hence, while proteins targeting other pathways may exhibit different patterns of diversification (Li et al., 2012), it is clear the antagonism between these DCL and anti‐*S. pneumoniae* antibodies is an important immune interface between host and pathogen.

## TARGETING THE IMMUNE INTERFACE

The activities of these DCL also resolves the paradox of why these proteins are so variable in bacteria expressing other conserved immunogenic proteins (Croucher et al., 2017): while the DCL proteins are active, antibodies targeting other antigens of the same cell are likely to be ineffective (Croucher et al., 2024). However, once the immune interface proteins are themselves inhibited by antibody binding, then it is likely the fully active polyvalent immune response can clear the bacteria from the host.

Therefore, a beneficial modification of the reverse vaccinology strategy may be to specifically target the bacterial proteins that enable evasion of antibody responses. Such formulations can be described as ‘immune interface interference’ (I3) vaccines. As such proteins must necessarily interact with host immune mediators, they will be accessible to vaccine‐induced responses, making them physiochemically ideal targets for all isotypes of immunoglobulins.

The challenge of I3 vaccine development will be targeting the diversity of immune interface proteins, which are likely to be under similar selection pressures as immunodominant viral proteins. However, the advent of genomic surveillance of pathogens makes it possible to identify the full species‐wide diversity of such loci (Blackwell et al., 2021). Using such resources to design panproteome arrays has revealed the natural expansion of host immunity eventually enables adults to recognise the full diversity of DCL proteins in *S. pneumoniae*, consistent with the immune system being capable of accumulating a broad polyvalent response to these antigens after a limited number of colonisation episodes (Croucher et al., 2024). Therefore, I3 vaccines should accelerate this maturation of the immune repertoire by broadening the set of antigens targeted by a child's immunity, rather than trying to strengthen existing responses to conserved proteins, as with immunisations designed by current reverse vaccinology approaches.

## VACCINES AGAINST TYPE B MENINGOCOCCI AS EXAMPLES OF I3 VACCINES

The first target of reverse vaccinology, serotype B meningococci, was selected because its capsule is composed of polysialic acid, which is found in human carbohydrates, and therefore cannot be targeted by pathogen‐specific immunity (Masignani et al., 2019). The analysis of an early genome sequence (Pizza et al., 2000) ultimately identified three protein antigens: NadA, NHBA and fHbp (Masignani et al., 2019). This trivalent formulation has subsequently been combined with outer membrane vesicles (OMVs), previously successfully used to control meningococcal outbreaks (Kelly et al., 2007), to generate the licensed vaccine Bexsero (Findlow et al., 2022). The second, independent, reverse vaccinology study identified a single antigen: fHbp (Fletcher et al., 2004). This design has since been expanded to a bivalent formulation, comprising two variants of fHbp, and licensed as Trumenba (Marshall et al., 2023). The fHbp protein on which both studies converged is the meningococcal Factor H‐binding protein, making it part of this bacterium's immune interface (Lo et al., 2009). Therefore, antibodies targeting fHBP should have a synergistic effect both in directly targeting this surface antigen and enhancing the activity of the host immune system (Principato et al., 2020).

The regulatory approval of these vaccines was based upon using immunological correlates of protection, rather than efficacy in preventing disease (Findlow et al., 2022). The ability of both formulations to induce a detectable response likely reflects the important trials being conducted in children (Gossger et al., 2012; Marshall et al., 2023), who are unlikely to have natural immunity to the specific fHbp variants included in the vaccines, given the diversity of this protein (Fletcher et al., 2004; Masignani et al., 2019) and the low pre‐adolescence carriage rate of meningococci (Christensen et al., 2010).

These vaccines will provide useful information on the potential of targeting all, or just some, mechanisms by which bacteria evade host defences, which will depend on the level of redundancy and interdependence in their functions. While Trumenba targets the broad diversity of a single variable component of the full immune interface (Lo et al., 2009), the inclusion of OMVs in Bexsero means it has some similarities with whole cell vaccines, effectively spanning the full immune interface of a subset of meningococcal diversity. Promisingly, vaccination of young infants with Bexsero in the UK appears to have had a positive impact in post‐licensure surveillance studies (Mensah et al., 2023; Parikh et al., 2016; Rodrigues et al., 2023).

## RECOMMENDATIONS FOR FUTURE VACCINE DESIGNS

Rather than the reverse vaccinology 2.0 approach of designing formulations to target conserved proteins, I3 vaccines would build on the original reverse vaccinology concept to use genomic data to target the full range of pathogen proteins that inhibit the host immune system. Hence, rather than aiming to quantitatively strengthen existing antibody responses, I3 vaccines would be intended to have a synergistic effect by both triggering responses to immunogenic proteins, and potentiating the host's natural immunity by debilitating the pathogen's capacity to inhibit host defences. Although challenging to design, we now have the molecular biology capacity to identify immune interface proteins and understand their sequence variation across populations. Therefore, I3 vaccines could enable new vaccine technologies (Jackson et al., 2020) to be applied to addressing the urgent challenge of increasingly difficult to treat bacterial infections (Frost et al., 2023).

## AUTHOR CONTRIBUTIONS

**Nicholas J. Croucher:** Conceptualization and writing.

## CONFLICT OF INTEREST STATEMENT

I have consulted for Antigen Discovery Inc. and Pfizer, been invited to attend meetings organised by Merck and have received an investigator‐initiated award from GlaxoSmithKline.
